# Correction: The eutectic point in choline chloride and ethylene glycol mixtures

**DOI:** 10.1039/d4cc90022g

**Published:** 2024-01-12

**Authors:** Hannah J. Hayler, Susan Perkin

**Affiliations:** a Physical and Theoretical Chemistry Laboratory, University of Oxford South Parks Road Oxford OX1 3QZ UK susan.perkin@chem.ox.ac.uk

## Abstract

Correction for ‘The eutectic point in choline chloride and ethylene glycol mixtures’ by Hannah J. Hayler *et al.*, *Chem. Commun.*, 2022, **58**, 12728–12731, https://doi.org/10.1039/D2CC04008E.

The authors regret that a problem in the experimental method led to some inaccurate data being published in the original article. During discussions at the recent 9th International Congress on Ionic Liquids in Lyon, it was pointed out by Dr Saffron Bryant (RMIT University, Australia) that the procedure described in the electronic supplementary information (ESI) for erasing the thermal history of the choline chloride : ethylene glycol mixtures was likely to have led to a degree of ethylene glycol loss and therefore an error in the mole fractions as reported in Fig. 2 and 3 of the original manuscript. The measurements of mass loss during the experiments have been revisited in order to try to quantify this error. Unfortunately, although there was indeed loss of mass during measurements, there were no systematic trends in the mass changes which would have allowed an adjustment or compensation for the error; this is likely due to small variations in the rate of evaporation between experiments carried out under similar cycling conditions due to differences in DSC pan hole dimensions. Nonetheless, it is highly likely that the reported eutectic composition range of 0.01 < *x*_ChCl_ < 0.02 is lower than the true eutectic composition. The authors believe that other conclusions from the manuscript remain robust, in particular the finding that the mixture of choline chloride with ethylene glycol is not a deep eutectic solvent. The statements in the manuscript about the problems associated with defining a substance by reference to its eutectic properties are ironically strengthened by this late discovery of an additional source of error. A recent manuscript by Meredith *et al.*^1^ reports an extensive and rigorous study of the thermodynamic properties of mixtures claimed to be deep eutectic solvents, to which readers are referred for a current and authoritative source of experimental evidence. Amongst many other findings, it is reported therein that the eutectic composition of choline chloride with ethylene glycol is in fact in the ratio of 1 : 3.

The Royal Society of Chemistry apologises for these errors and any consequent inconvenience to authors and readers.

## Supplementary Material
